# The Impact of Pipe Material on the Diversity of Microbial Communities in Drinking Water Distribution Systems

**DOI:** 10.3389/fmicb.2021.779016

**Published:** 2021-12-21

**Authors:** Debbie Lee, Gennaro Calendo, Kristin Kopec, Rebekah Henry, Scott Coutts, David McCarthy, Heather M. Murphy

**Affiliations:** ^1^Water, Health and Applied Microbiology Laboratory (WHAM Lab), Department of Epidemiology and Biostatistics, College of Public Health, Temple University, Philadelphia, PA, United States; ^2^Environmental and Public Health Microbiology Laboratory (EPHM Lab), Department of Civil Engineering, Monash University, Clayton, VIC, Australia; ^3^Micromon, Department of Microbiology, Monash University, Clayton, VIC, Australia; ^4^Water, Health and Applied Microbiology Laboratory (WHAM Lab), Department of Pathobiology, Ontario Veterinary College, University of Guelph, Guelph, ON, Canada

**Keywords:** pipe material, annular reactor, drinking water, microbiome, biofilm

## Abstract

As many cities around the world face the prospect of replacing aging drinking water distribution systems (DWDS), water utilities must make careful decisions on new pipe material (e.g., cement-lined or PVC) for these systems. These decisions are informed by cost, physical integrity, and impact on microbiological and physicochemical water quality. Indeed, pipe material can impact the development of biofilm in DWDS that can harbor pathogens and impact drinking water quality. Annular reactors (ARs) with cast iron and cement coupons fed with chloraminated water from a municipal DWDS were used to investigate the impact of pipe material on biofilm development and composition over 16 months. The ARs were plumbed as closely as possible to the water main in the basement of an academic building to simulate distribution system conditions. Biofilm communities on coupons were characterized using 16S rRNA sequencing. In the cast iron reactors, β-proteobacteria, Actinobacteria, and α-proteobacteria were similarly relatively abundant (24.1, 22.5, and 22.4%, respectively) while in the cement reactors, α-proteobacteria and Actinobacteria were more relatively abundant (36.3 and 35.2%, respectively) compared to β-proteobacteria (12.8%). Mean alpha diversity (estimated with Shannon H and Faith’s Phylogenetic Difference indices) was greater in cast iron reactors (Shannon: 5.00 ± 0.41; Faith’s PD: 15.40 ± 2.88) than in cement reactors (Shannon: 4.16 ± 0.78; Faith’s PD: 13.00 ± 2.01). PCoA of Bray-Curtis dissimilarities indicated that communities in cast iron ARs, cement ARs, bulk distribution system water, and distribution system pipe biofilm were distinct. The mean relative abundance of *Mycobacterium* spp. was greater in the cement reactors (34.8 ± 18.6%) than in the cast iron reactors (21.7 ± 11.9%). In contrast, the mean relative abundance of *Legionella* spp. trended higher in biofilm from cast iron reactors (0.5 ± 0.7%) than biofilm in cement reactors (0.01 ± 0.01%). These results suggest that pipe material is associated with differences in the diversity, bacterial composition, and opportunistic pathogen prevalence in biofilm of DWDS.

## Introduction

According to the American Academy of Microbiology, “the distribution system is the remaining component of public water supplies yet to be adequately addressed in national efforts to eradicate waterborne disease” (Ingerson-Mahar and Reid, 2012). There is ongoing debate regarding whether water distribution networks contribute a measurable health risk derived from pathogens which can intrude into or colonize these systems. The Safe Drinking Water Act (SDWA) regulates the quality of drinking water from every public water system in the United States and thus, municipal drinking water is often presumed to be free of microbial contaminants. However, biofilm inevitably develops and can persist on the inner surfaces of pipe in drinking water distribution systems (DWDS) through the formation of a matrix of extracellular polymeric substances, which provide physical and chemical support for biofilm (Flemming et al., 2002; Flemming and Wingender, 2010). Most of the bacteria present within DWDS is estimated to be housed within biofilm lining these pipe and not in the bulk water (Flemming et al., 2002; Liu G. et al., 2014). Biofilm are composed of numerous, often innocuous, organisms, but some may be pathogenic (Lehtola et al., 2007; Wingender and Flemming, 2011). The United States Centers for Disease Control and Prevention (CDC) recently attributed most hospitalizations and deaths from waterborne illness to biofilm-associated pathogens, such as non-tuberculous mycobacteria, *Pseudomonas*, and *Legionella* (Collier et al., 2021). When biofilm-associated pathogens are mobilized, which may occur for a number of reasons, including during changes to flow increasing shear stress in the system, there is increased concern for vulnerable populations, including children, the elderly, and the immunocompromised. Yet, the drivers of biofilm development and composition in drinking water distribution systems are still not well understood (Ingerson-Mahar and Reid, 2012).

Increased accessibility of sequencing has made possible a greater characterization of drinking water biofilm in both model (pilot and laboratory-based) and full-scale systems (Chao et al., 2015; Lührig et al., 2015; Proctor et al., 2017; Perrin et al., 2019; Cruz et al., 2020; Li et al., 2020). Biofilm development and composition on the inner surfaces of pipe may be driven by disinfectant residual, water temperature, total or assimilable organic carbon, and pipe material (Douterelo et al., 2014; Wang et al., 2014; Proctor et al., 2017; Aggarwal et al., 2018). Better understanding the impact of pipe material on biofilm is a growing need as many aging distribution systems look to replace old cast iron networks with pipe composed of other materials, such as PVC or cement-lined ductile iron pipe, or retrofitting existing pipe with cured in place pipe. Pipe materials may differ in their support of microbial community development, which in turn, may differentially promote the proliferation of pathogens within biofilm. To date, biofilm development on different pipe materials in conditions representative of distribution systems is still largely uncharacterized (Ingerson-Mahar and Reid, 2012).

Studies of the impacts of pipe material on biofilm formation often employ bench-scale, laboratory-based reactors, which provide the opportunity to study biofilm under controlled conditions (Gomes et al., 2014). Prior laboratory-based work has found differences in the composition of biofilm depending on pipe material (e.g., PVC, copper, iron) (Buse et al., 2014; Wang et al., 2014; Aggarwal et al., 2018). Using annular reactors (ARs) plumbed into the distribution system of a major metropolitan city in the northeastern United States, the present study aims to compare the diversity of and differences in biofilm composition according to pipe material and physicochemical water quality parameters at a novel temporal scale (16-month duration).

The present study is unique as it plumbed ARs as close to the water main as possible instead of running experiments in a laboratory setting, so that reactor influent was largely unimpacted by the building plumbing with high chlorine levels and reactors were more likely to be representative of the DWDS. To our knowledge, no study has plumbed ARs at the entry point of a building to more closely emulate the *in situ* conditions of a DWDS. Annular reactor experiments are typically conducted in laboratory settings using water influenced by premise plumbing, which has distinct impacts on resulting water quality due to myriad factors, including the types of pipe used within the building (which differ from what is in the distribution system), longer retention times, temperature differences, and disinfectant decay (Lautenschlager et al., 2010; Nguyen et al., 2012; Douterelo et al., 2013; Ji et al., 2015; Ling et al., 2018).

Furthermore, no biofilm studies have sampled at the frequency (weekly for most of the study period) and few have sampled for the same duration (up to 16 months) of the present study. Frequent sampling was selected to examine the temporal variation in biofilm development in terms of microbial communities using 16S sequencing and to fill a gap in knowledge on early biofilm development. Biofilm composition can change quickly during the early stages of development and thus, frequent assessments of biofilm are necessary to assess the composition of rapidly changing biofilm.

## Materials and Methods

### Annular Reactor Setup and Sample Collection

Six rotating annular reactors (ARs) (Model 1320 LS, BioSurface Technologies Corporation, Bozeman, MT, United States) were installed in the basement of a building sourcing chloraminated water from a large DWDS in the northeastern United States that uses conventional water treatment and sources water from a major river. Water age at the building location is typically less than 3 days. These ARs were plumbed as proximal to the point of entry of water in the building to best approximate *in situ* conditions of the DWDS and avoid the impacts of premise plumbing on biofilm development. Three ARs (AR 1, 2, 3) each contained 20 pitted, rusted, visibly aged cast iron coupons (Supplementary Figure 1; 19.5 cm^2^ surface area; BioSurface Technologies Corporation, Bozeman, Montana) and the other three ARs (AR 4, 5, 6) each contained 20 cement coupons. Cast iron coupons were purchased 10 years before experiments were conducted and used in other experiments for 3 years. Cement coupons were new at the start of these experiments. Once set up, ARs were run in parallel for 6 weeks (conditioning period) to allow the development of a steady state biofilm within the inner drum (Camper, 1995; Manuel et al., 2007). Reactors were operated at a rotational speed of 50 rpm, corresponding to shear stress of 0.25 N/m^2^ at the outer wall, similar to shear stress conditions of other laboratory-based investigations of distribution systems (Gagnon et al., 2005; Murphy et al., 2008). The reactors had a hydraulic retention time (HRT) of 2 h, corresponding to a flow rate of 7.9 mL/min, which simulates zones of shorter water age within the distribution system.

Following the initial 6-week conditioning period, one coupon from each set (cast iron or cement) was sampled aseptically (using sterile hook and clamp) weekly for the first 34 weeks and then monthly for four additional months (cast iron ARs) and six additional months (cement ARs). Weekly and monthly coupons were randomly pulled from a different reactor in each set (cast iron or cement) rotating through each set in sequential order (AR 1 and AR 4, AR 2 and AR 5, AR 3 and AR 6). Only one reactor of each type was sampled at each timepoint. Biofilms were scraped five times from each coupon’s surface using a sterile knife and then suspended in 25 mL of 0.1% Tween 80 solution and 20 μL of 10% w/v sodium thiosulfate (Chao et al., 2015). A total of 78 coupons (38 cast iron; 40 cement) were collected over 16 months (June 2017 to October 2018; no biofilm samples collected June/July 2017). Cast iron coupons were collected over only 14 months (June 2017 to August 2018) due to coupon corrosion and infeasibility of coupon removal from ARs.

On March 5, 2018, nine biofilm samples were also collected from the biofilm of a cast iron main during a water main break (Supplementary Figure 2). The main, installed in 1860, was 25.4 cm in diameter, received water from two major rivers, and on an average day, had a flow rate of 8.8 × 10^–4^ cm and velocity of 1.5 × 10^–2^ m/s. Pipe samples were collected by either scraping the interior of the pipe using a sterile knife or by breaking off pieces of the pipe. The scraped material or pipe pieces were suspended in the Tween solution previously described (Supplementary Figure 3). The pipe material of the aged cast iron coupons was similar to the segments retrieved during this main break (pictures of pipe segments can be found in Supplementary Figure 3). Samples were vacuum-filtered with mixed cellulose ester membrane filters with a pore size of 0.22 μm (MF-Millipore, Darmstadt, Germany). An additional 437.6 L bulk water sample was also collected from the backflow into the building following this main break using dead-end ultrafiltration with a REXEED 25s hemodialyzer filter (Asahi Kasei Medical MT Corp., Oita, Japan). The bulk water sample was collected after flushing the tap until water was no longer discolored (due to the main break). Flushing was done to ensure the water sample collected was representative of what communities served by this DWDS would consume. Ultrafilter elution and secondary concentration were performed using methods previously described (Smith and Hill, 2009). The biofilm elutions and bulk water concentrate were used for sequencing analysis (described in section “DNA Extraction and Sequencing”).

The heterotrophic plate count (HPC) of biofilm on AR coupons was measured by plating serial dilutions of biofilm elutions and bulk water samples on R2A agar (Remel, San Diego, CA, United States), following Standard Method 9215C (Eaton et al., 1995). The local water utility provided real-time measurements of total chlorine, conductivity, temperature, and turbidity at 5-min intervals using sensors installed at the experimental site of the influent water. Weekly water samples collected from the ARs were also monitored for total chlorine, conductivity, temperature, turbidity, and pH.

### DNA Extraction and Sequencing

DNA from biofilm elutions and bulk water concentrates (collected following the main break) were extracted using the MoBio DNA Extraction kit (Qiagen, Hilden, Germany). DNA extractions of autoclaved, deionized water served as negative controls. Sequencing libraries were prepared at Micromon Genomics (Monash University, Clayton, VIC, Australia) from the V3-V4 region (Primer sequences can be found in Supplementary Text 1) of the prokaryotic 16S rRNA gene, according to the Illumina *16S Metagenomics Sequencing Library Preparation* guide with the following modifications: briefly, 3 μL of genomic DNA was used as a template to produce primary amplicons after subjecting the reactions to 20 cycles of PCR. Amplicons were sequenced using Illumina MiSeq v3 (Micromon, Monash University, Australia), with the PhiX Control Library V3 (Illumina Inc., San Diego, CA, United States) as a spike-in control. Indexes were added using a Nextera XT Indexing Kit (Illumina Inc., San Diego, CA, United States) using 10 PCR cycles. Purified final amplicons were diluted to 4.75 pM, denatured and sequenced at Micromon Genomics (Monash University, Clayton, VIC, Australia) as per the manufacturer’s instructions using Illumina MiSeq with a MiSeq Reagent Kit V3 (Illumina Inc., San Diego, CA, United States). Negative control libraries were constructed with ultrapure water as the input material. These libraries produced no measurable product and were removed from further processing.

### Sequence Data Analysis

The resulting demultiplexed, raw paired-end reads were imported into QIIME2 (Bolyen et al., 2019) and CutAdapt was used to remove primers (Martin, 2011). Divisible Amplicon Denoising Algorithm 2 (DADA2) was used to filter, trim, identify chimeras in, and denoise the data and merge paired-end reads (Callahan et al., 2016). Amplicon sequence variant (ASV) tables were filtered to remove ASVs that were not classified beyond the phylum level, occurred in only one sample, or identified as contaminants by comparing samples with negative controls using the decontam package in R (Davis et al., 2018).

The representative sequences for each ASV were used to construct a *de novo* phylogenetic tree. Sequences were aligned using MAFFT and then masked to filter highly gapped regions in the alignment (Katoh and Standley, 2013). Phylogenies were inferred using IQ-TREE with the ultrafast bootstrap method (Minh et al., 2013; Nguyen et al., 2014; Kalyaanamoorthy et al., 2017). The resulting phylogenetic tree was midpoint-rooted. Taxonomic classification of the representative sequences was performed by a Naïve–Bayes classifier trained on the SILVA database (release 132; accessed December 5, 2019) (Quast et al., 2012).

### Statistical Analyses

To obtain even sampling depth for diversity analyses, samples were rarefied to depths of 34,585 reads and all samples were retained at this level. Alpha and beta diversity indices were estimated using the q2-diversity plugin in QIIME2. Alpha diversity was estimated using Shannon H index and Faith’s Phylogenetic Diversity (PD). Beta diversity was estimated using Bray-Curtis dissimilarities and weighted UniFrac distances. Principal Coordinate Analysis (PCoA) on distance matrices was performed using the same plugin.

To assess which taxa were differentially abundant according to pipe material, longitudinal ANCOM was performed using the ANCOM2 package in R. Features with w-values greater than 0.7 were considered differentially abundant (Mandal et al., 2015). The trendyspliner function of the SplinectomeR package in R was used to analyze alpha diversity over time (Shields-Cutler et al., 2018). Using the vegan package in R (Oksanen et al., 2018), comparisons of beta diversity across cast iron and cement ARs were performed using Permutational Multivariate Analysis of Variance (PERMANOVA) with 999 permutations using the adonis function and the homogeneity of group dispersions were analyzed using the betadisper function. Correlations between physicochemical parameters and alpha diversity metrics were examined using pairwise Spearman rank correlations.

The presence of putative and opportunistic pathogenic species was examined by querying BLAST with the representative sequences of the ASVs of clinically relevant genera. The PICRUSt2 plugin for QIIME2 was used to infer the functional composition of samples (Douglas et al., 2019). Differences in the relative abundance of functional and metabolic pathways (MetaCyc and KEGG Orthology) according to pipe material, season, and physicochemical parameters were assessed using Kruskal–Wallis and Dunn tests. In particular, the relative abundance of genes involved in MetaCyc pathways and KEGG Orthology pathways associated with nitrogen metabolism (e.g., nitrification, ammonia oxidation) were briefly investigated (Supplementary Texts 2, 3).

## Results

### Water Quality Parameters

**FIGURE 1:**
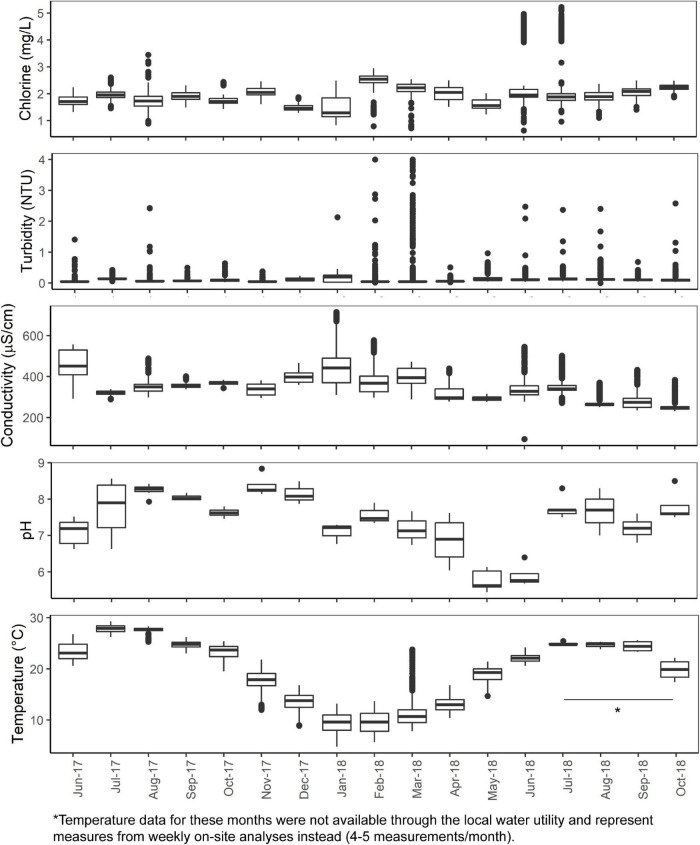
Total chlorine, turbidity, temperature, conductivity (5-min data provided by the local water utility) and pH and temperature (analyzed weekly on-site) of the AR influent water from June 2017 to October 2018.

The mean total chlorine, conductivity, turbidity, and temperature levels of the effluent water from the cast iron and cement ARs were similar (*p* > 0.05) though mean chlorine levels trended higher in the cement ARs and mean turbidity trended higher in the cast iron ARs. A summary of the mean total chlorine, conductivity, turbidity, and temperature levels of the influent water and of the effluent water (collected from the ARs) over the course of the study can be found in Figure 1 and Table 1. Influent water (data from the local water utility) generally maintained mean chlorine levels of 1.94 ± 0.48 mg/L but in some instances, reached lows of 0 mg/L and highs of 5.23 mg/L. Influent water temperature fluctuated seasonally, with a minimum of 7.6°C and a maximum of 27.9°C.

**TABLE 1:** Summary of microbiological and physicochemical water quality in annular reactor (AR) influent (5-min data provided by local water utility over the study period) and effluent (analyzed weekly over the study period).

	AR Influent Mean ± Standard Deviation	Cast Iron AR Effluent Mean ± Standard Deviation	Cement AR Effluent Mean ± Standard Deviation
HPC	1.54 ± 6.29 × 10^7^CFU/100 mL	1.05 ± 1.16 × 10^8^CFU/cm^2^	2.60 ± 7.47 × 10^7^CFU/cm^2^
Total chlorine	1.94 ± 0.48 mg/L	0.16 ± 1.35 mg/L	0.40 ± 0.40 mg/L
Conductivity	331.77 ± 51.33 μS/cm	324.78 ± 40.82 μS/cm	325.98 ± 55.17 μS/cm
Temperature	20.59 ± 5.65°C	24.50 ± 3.65°C	24.64 ± 2.52°C
Turbidity	0.85 ± 1.09 NTU	1.24 ± 1.38 NTU	0.89 ± 0.73 NTU
pH	7.49 ± 0.86	7.41 ± 0.73	7.32 ± 0.77

*CFU, colony forming unit; NTU, nephelometric turbidity unit. μS/cm, microsiemens per centimeter.*

HPC concentrations were significantly different between the cast iron and cement reactors (Kruskal–Wallis χ^2^ = 19.9; *p* < 0.001). Concentrations were higher in the cast iron reactors (1.05 ± 1.16 × 10^8^CFU/cm^2^) than in the cement reactors (2.60 ± 7.47 × 10^7^CFU/cm^2^).

### Relative Abundance of Taxa Differ by Pipe Material

All ASVs (from ARs, distribution system pipe biofilm, distribution system water samples) were assigned to 1,853 taxa. Of these, 581 taxa were from the AR samples. Nearly 73% of these taxa were low in relative abundance (<1%) across all AR samples.

In the cast iron reactors, β-proteobacteria, Actinobacteria, and α-proteobacteria were similarly relatively abundant (24.1%, 22.5%, and 22.4%, respectively) while in the cement reactors, α-proteobacteria and Actinobacteria were more relatively abundant (36.3 and 35.2%, respectively) compared to β-proteobacteria (12.8%).

**FIGURE 2:**
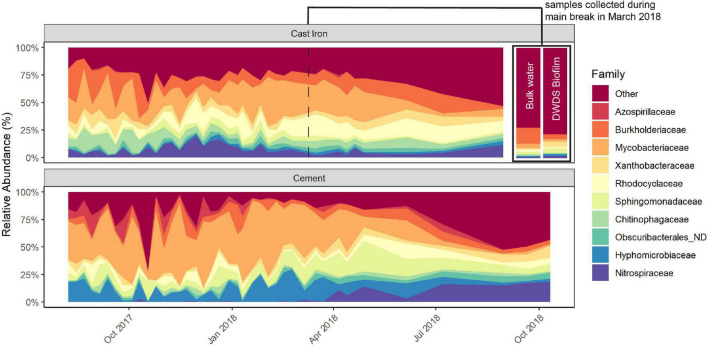
Relative abundance of the most prevalent taxa in cast iron and cement annular reactor (AR) biofilm samples over the 16-month study period. At each time point, one coupon was collected from the set of cast iron ARs and the set of cement ARs. One coupon from each set was collected weekly for the first 34 weeks and then monthly for four additional months (cast iron ARs) and six additional months (cement ARs). A total of 38 coupons from the cast iron ARs and a total of 40 coupons from the cement ARs were collected. The relative abundance of the top AR taxa in bulk water and drinking water distribution system (DWDS) pipe biofilm collected during the main break in March 2018 can be found in the top right section.

The predominant families in the cast iron biofilm were *Mycobacteriaceae* (21.7%), *Burkholderiaceae* (12.8%), and *Rhodocyclaceae* (10.0%). In the cement biofilm, *Mycobacteriaceae* (34.8%), *Hyphomicrobiaceae* (11.3%), and *Sphingomonadaceae* (9.2%) were the most relatively abundant. The relative abundance of these predominant families remained similar throughout the study but most notably, the relative abundance of *Mycobacteriaceae* diminished in both cast iron and cement reactors and *Nitrospiraceae* increased in both types of reactors (Figure 2 and Supplementary Figure 4).

**FIGURE 3:**
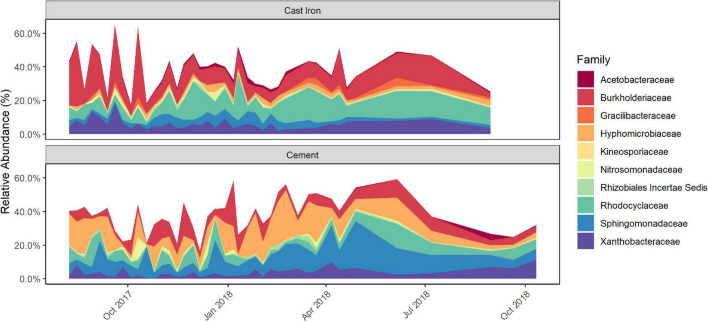
Relative abundance of differentially abundant taxa in cast iron and cement annular reactor (AR) biofilm samples over the 16-month study period as identified by Longitudinal Analysis of Composition of Microbiomes (ANCOM) analysis. At each time point, one coupon was collected from the set of cast iron ARs and the set of cement ARs. One coupon from each set was collected weekly for the first 34 weeks and the monthly for four additional months (cast iron ARs) and six additional months (cement ARs). A total of 38 coupons from the cast iron ARs and a total of 40 coupons from the cement ARs were collected.

Longitudinal ANCOM results indicated that the families that were differentially abundant by pipe material were: *Acetobacteraceae*, *Burkholderiaceae*, *Gracilibacteraceae*, *Hyphomicrobiaceae*, *Kineosporiaceae*, *Nitrosomonadaceae*, *Rhizobiales Incertae Sedis*, *Rhodocyclaceae*, *Sphingomonadaceae*, and *Xanthobacteraceae*. *Burkholderiaceae*, *Rhodocyclaceae*, *Hyphomicrobiaceae*, and *Sphingomonadaceae* (Figure 3 and Supplementary Figure 5) were—with W-statistics greater than 0.9—the most statistically differentially abundant families between the types of pipe material.

### Alpha Diversity in Cast Iron and Cement Reactors Over Time and by Season

**FIGURE 4:**
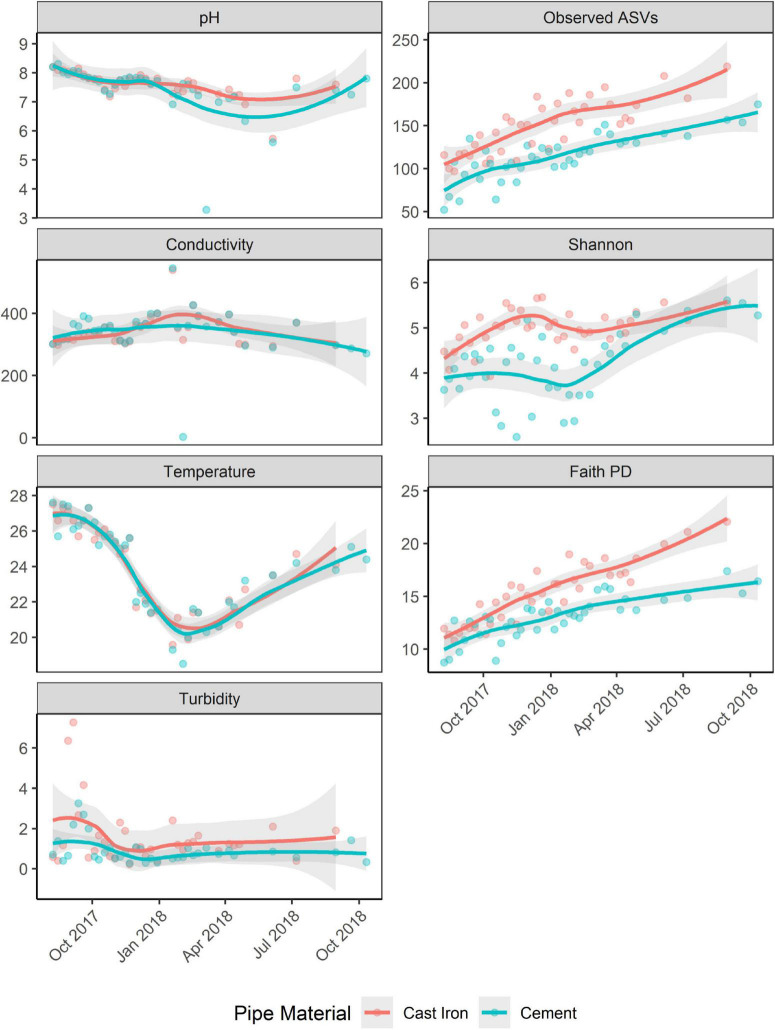
Observed ASVs, Faith PD, and Shannon H indices of biofilm samples from annular reactors (ARs) and physicochemical parameters [pH, temperature (Celsius), conductivity (pS/cm), and turbidity (NTU)] of water samples collected from ARs during coupon collection. For the first 34 weeks, one cast iron coupon and one cement coupon were collected. Then, one cast iron coupon was collected monthly for four more months and one cement coupon was collected monthly for six more months. Loess method used to fit the smooth curve. Gray bands represent the 95% confidence interval.

Richness (Observed ASVs) and diversity (Faith’s PD, Shannon) generally increased over the course of the study (Figure 4). Alpha diversity across both sample types increased over time (trendyspliner; *p* = 0.001).

Shannon H indices of ARs were not significantly associated with season. Faith’s PD was significantly associated with season (Kruskal–Wallis χ^2^ = 17.89; *p* < 0.001). *Post hoc* Dunn’s test showed significant differences in Faith’s PD between spring and autumn, spring and summer, and winter and autumn (Bonferroni corrected; *p* < 0.05).

Pipe material was associated with differences in diversity (Shannon and Faith’s PD) and richness (Observed ASVs). Alpha diversity in the cast iron reactors was significantly greater (Kruskal–Wallis χ^2^; *p* < 0.001 for Shannon H indices, Faith PD, and Observed ASVs) than alpha diversity in cement reactors (Supplementary Table 1).

### Correlation Between Bacterial Diversity and Physiochemical Parameters

There was no significant correlation between Shannon H indices and maximum temperature and turbidity in the month preceding sampling. There was a negative correlation between Shannon indices and total chlorine levels (Spearman’s rho = −0.25, *p* = 0.03) and Shannon indices and conductivity (Spearman’s rho = −0.28, *p* = 0.01).

There was a negative correlation between Faith PD and maximum and mean temperature in the month preceding sampling (maximum temperature: Spearman’s rho = −0.47, *p* < 0.001; mean temperature: Spearman’s rho = −0.50, *p* < 0.001). Positive correlations were observed between Faith PD and mean total chlorine (Spearman’s rho = 0.27, *p* = 0.02) and Faith PD and mean turbidity (Spearman’s rho = 0.35, *p* = 0.001) in the month preceding sampling. A detailed table of correlations between Shannon indices, Faith PD, and Observed ASVs can be found in Supplementary Table 2.

### Distinct Communities Developed in Cast Iron and Cement Reactors

While the most prevalent taxa in the AR samples were also found in the distribution system water and distribution system pipe biofilm samples (Supplementary Figure 6), the distribution system water (collected once) and distribution system pipe biofilm samples (collected in 1 day) following the main break, contained numerous other taxa not represented in the AR samples.

**FIGURE 5:**
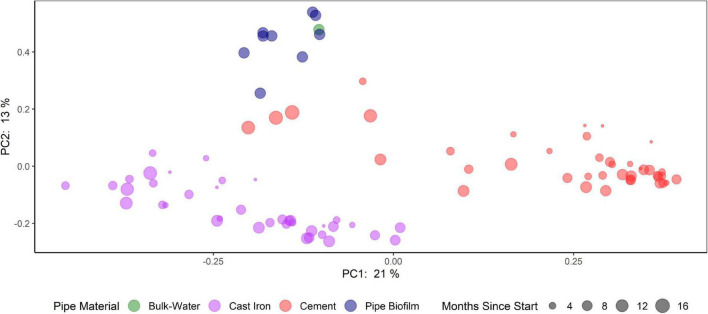
Comparison of bacterial communities in annular reactors (cast iron and cement) and drinking water distribution systems (bulk water and pipe biofilm). Principal coordinate analysis (PCoA) plots of the Bray-Curtis dissimilarities according to their sample type (color) and months since start of study (size).

Principal Coordinate Analysis (PCoA) using Bray-Curtis dissimilarities (Figure 5 and Supplementary Figure 7) suggest that bacterial communities clustered according to pipe material and sample type (cast iron AR, cement AR, distribution system water, distribution system pipe biofilm). Samples from the distribution system pipe biofilm and distribution system water also clustered together and these distribution system samples differed from the AR samples. PCoA of weighted UniFrac distances suggest that when considering phylogenetic similarity, clustering by pipe material and sample type, while observed, was less distinct (Supplementary Figure 8).

Pipe material influenced variations in the beta diversity of the ARs (PERMANOVA: *R*^2^ = 0.21, *p* = 0.001). Additionally, variations in beta diversity were observed by season (PERMANOVA: *R*^2^ = 0.10, *p* = 0.001). Homogeneous dispersion was observed by pipe material (*p* = 0.34) but not by season (*p* = 0.001). Results of PERMANOVA and permutation tests of the homogeneity of dispersion can be found in Supplementary Table 3.

### Identified Opportunistic Pathogens

Through BLAST queries of representative sequences from the ASVs, opportunistic pathogens, such as *Mycobacterium avium* [26% (*n* = 20) of AR samples], were identified in AR biofilm and distribution system pipe biofilm samples as well as a distribution system water sample (Table 2). Some species were not identified in multiple sample types but were limited to only one sample type, either the distribution system water, distribution system pipe biofilm, or AR samples; however, some species (e.g., *Mycobacterium abscessus*) were identified in both the distribution system and AR biofilm samples.

**TABLE 2:** Identified opportunistic pathogens in annular reactor (AR; cast iron or cement substrata) biofilm, distribution system (DS) pipe biofilm, and bulk water (distribution system) samples.

Genera	Sample Type	Identified Opportunistic Pathogens
*Acinetobacter*	Bulk water	*Acinetobacter baumanii*
	AR [cast iron (1)]	*Acinetobacter lwoffii*
*Enterococcus*	Bulk water	*Enterococcus faecalis*
*Legionella*	AR [cast iron (19), cement (5)]; DS pipe (1)	*Legionella hackeliae*
*Mycobacterium*	AR [cast iron (37), cement (34)]; DS pipe (2)	*Mycobacterium abscessus*
	AR [cast iron (30), cement (29)]; bulk water; DS pipe (1)	*Mycobacterium boenickei*
	AR [cast iron (13), cement (7)]	*Mycobacterium avium*
	DS Pipe (3)	*Mycobacterium doricum*
	AR [cast iron (13), cement (9)]; bulk water; DS pipe	*Mycobacterium gordonae*
	AR [cast iron (38), cement (39)]; bulk water; DS pipe (8)	*Mycobacterium llatzerense*
	Bulk water; DS pipe (6)	*Mycobacterium lentiflavum*
	AR [cast iron (12), cement (5)]	*Mycobacterium kansasii*
	AR [cast iron (1), cement (4)]	*Mycobacterium marseillense*
	AR [cement (5)]	*Mycobacterium mucogenicum*
	AR [cast iron (6), cement (18)]; bulk water; DS pipe (9)	*Mycobacterium paragordonae*
	AR [cast iron (13), cement (1)]	*Mycobacterium parascrofulaceum*
	Bulk water; DS pipe (2)	*Mycobacterium rhodesiae*
	AR [cast iron (2), cement (3)]	*Mycobacterium saskatchewanense*
	AR [cast iron (13), cement (28)]; DS pipe (1)	*Mycobacterium smegmatis*
	AR [cast iron (3)]	*Mycobacterium shinjukuense*
*Pseudomonas*	AR [cast iron (1)]	*Pseudomonas mendocina*
	AR [cast iron (3), cement (3)]	*Pseudomonas putida*

*Number of samples where the opportunistic pathogen was detected is provided in parentheses.*

### *Legionella* spp. and *Mycobacterium* spp. Presence Differs in Cast Iron and Cement Reactors

The mean relative abundance of *Mycobacterium* spp. was greater in the cement reactors (34.8 ± 18.6%) than in the cast iron reactors (21.7 ± 11.9%). In contrast, the mean relative abundance of *Legionella* spp. trended higher in biofilm from cast iron reactors (0.5 ± 0.7%) than biofilm in cement reactors (0.01 ± 0.01%). Relative abundances of *Mycobacterium* spp. and *Legionella* spp. significantly differed by pipe material (*p* < 0.001 for *Legionella*, *p* = 0.001 for *Mycobacterium*). Relative abundances of *Mycobacterium* spp. significantly differed by season (*p* = 0.02), with relative abundance in this order: winter > autumn > summer > spring. Relative abundances of *Legionella* spp. did not significantly differ by season (*p* = 0.40) with relative abundance in this order: autumn > winter > spring/summer.

## Discussion

The present study found that biofilm of both cast iron and cement ARs were predominantly composed of the classes: α-proteobacteria, β-proteobacteria, and Actinobacteria. These major taxa are similar to the classes identified in other studies of biofilm and mobilized materials within chlorinated DWDS (Henne et al., 2012; Douterelo et al., 2014; Fish and Boxall, 2018). Proteobacteria and Actinobacteria have also been identified as predominant members of biofilm of chloraminated systems (Bal Krishna et al., 2013; Ling and Liu, 2013; Revetta et al., 2016).

### Biofilm Diversity, Pathogen Presence, and Pipe Material

The findings of the present study suggest that, compared to cement pipe, cast iron pipe are associated with greater heterotrophic plate counts and more diverse biofilm. Prior work has shown that, compared to other pipe materials, the cast iron substratum often promotes the development of more biomass (Niquette et al., 2000; Zhu et al., 2014) and greater diversity (Lin et al., 2013). It has been suggested that this may be because of the rough texture of the cast iron compared to other pipe materials, which has greater surface area and facilitates greater adhesion (Kerr et al., 1998), or differential nutrient availability (e.g., phosphorus release during corrosion) on cast iron pipe promoting bacterial growth (Morton et al., 2005; Douterelo et al., 2016). Increased corrosion of iron pipe has also been shown to limit the effectiveness of disinfectants in biofilm control (LeChevallier et al., 1993; Wang et al., 2012), which may contribute further to the greater development of biofilm on cast iron pipe.

It is important to note though that the impacts of increased growth and diversity of biofilm on drinking water quality are complex. On one hand, highly diverse and dense biofilm increase chlorine demand and may be more resistant to disinfection—multispecies biofilm (compared to single-species biofilm) exhibit greater resistance to disinfection (Elvers et al., 2002; Burmølle et al., 2006; Simões et al., 2010). While studies of the impacts of single vs. multi-species biofilm may not be directly comparable to studies of more modest differences in the diversity of multi-species biofilm, it is possible that increased diversity may help organisms survive in biofilm. Once transient microbes are incorporated into biofilm, it is possible that the greater diversity may protect, and promote the growth, these microbes by allowing them to reap the benefits of being part of a vast, synergistic biofilm network (Murga et al., 2001; Flemming and Wingender, 2010). This is the case for *L. pneumophila*, which can better adhere to rough biofilm surfaces with high surface area (Shen et al., 2015) and is well-adapted to persist in multispecies biofilm, even when certain species antagonistic to *L. pneumophila* are present (Stewart et al., 2012; Boamah et al., 2017). This characteristic accords with the results from the present study, which found a greater relative abundance of *Legionella* spp. in the more diverse cast iron samples (vs. cement samples).

However, increased diversity may also result in the increased resistance of the existing biofilm to transient microbes and perhaps pathogen colonization (Da Re et al., 2013). It has been demonstrated that extant bacteria within biofilm can resist the invasion of other microorganisms through synergistic interactions, the release of antagonistic compounds, and competition (Burmølle et al., 2006; Guerrieri et al., 2008; Rendueles and Ghigo, 2015; Nadell et al., 2016). It is thereby possible that the greater diversity of microbial communities observed on cast iron substratum (as observed in the present work) may potentially limit the growth of transient microbes, which may include opportunistic pathogens, introduced into DWDS. This relationship between biofilm diversity and opportunistic pathogen presence may help explain the lower relative abundance of mycobacteria in the cast iron samples of the present study compared to the relative abundance in the cement samples, which had lower HPC levels and microbial diversity. The greater abundance of mycobacteria in cement (vs. cast iron) samples was also identified in another study of opportunistic pathogens in biofilm (Wang et al., 2012), in which the authors posited that iron pipe may be more conducive to the proliferation of other bacteria, thereby limiting the relative abundance of *Mycobacterium* spp.

Aside from the impacts of biofilm diversity, differences in corrosion products from different pipe materials may also impact pathogen presence in drinking water biofilm. In a recent study, Haig et al. (2020) found that dissolved iron levels in water were positively associated with the abundance of specific opportunistic pathogens and genera containing opportunistic pathogens, such as non-tuberculous mycobacteria. While research on the impact of pipe corrosion and pathogen survival is limited, Fu et al. (2021) recently found that pipe corrosion and corrosion products were associated with the enhanced survival of pathogens in drinking water distribution systems.

Beta diversity analyses indicated that microbial composition differed significantly between distribution system water and biofilm samples. It is important to note that only one bulk distribution system water sample (after main break) was collected in the present study but this difference between the composition of distribution system water and biofilm has been noted in previous studies (Henne et al., 2012; Liu G. et al., 2014). Future work should investigate the impact of biofilm diversity on pathogen presence in DWDS pipe biofilm but also pathogen presence in bulk water from DWDS.

### Study Strengths, Caveats and Future Research Recommendations

The present study has several strengths and represents an innovative contribution to our understanding of biofilm development and composition within DWDS pipe over time. Laboratory-based studies are often performed over either a short period of time (Fish et al., 2015) or more sporadically, over long periods of time and thus the combination of the frequency (weekly in the beginning) and duration (16 months) of sampling allowed this study to elucidate some of the dynamics of biofilm composition. These results suggest that in the early stages of biofilm development within pipe, the relative abundance of taxa is constantly shifting. Another strength of this study was the installation of the annular reactors at the point of water entry in the building. The relatively low water age (<3 days) and high disinfectant levels of water at our study location suggest that the ARs were representative of the drinking water distribution system. This allowed ARs to receive influent water not influenced by premise plumbing conditions and thereby better approximate the natural, *in situ* conditions of the DWDS. Annular reactor experiments rarely employ unadulterated water proximal to a DWDS main, which complicates the interpretation of biofilm development within the reactors. Furthermore, a main break that occurred during the experimental period provided the opportunity to compare annular reactor biofilm to distribution system pipe biofilm.

Additionally, the results of the present work are generalizable to many other public water systems in the United States. Similar to many other systems across the country, the DWDS associated with this study employs conventional water treatment methods, disinfects with chloramines [United States Environmental Protection Agency (USEPA), 2009a], relies on surface water sources [United States Environmental Protection Agency (USEPA), 2009b], experiences both warm and cold temperature conditions, and is composed of networks of predominantly cast iron and cement-lined ductile iron pipe [American Water Works Association (AWWA), 2017].

However, there are important caveats to the interpretation of the present study’s results. For one, the temperature in the ARs was higher and less variable (cast iron mean: 24.50 ± 3.65°C; cement mean: 24.64 ± 2.52°C) than the temperature in the influent water (20.59 ± 5.65°C). The impact of pipe material on biofilm composition and development may differ depending on water temperature (Proctor et al., 2017). The higher temperature in the ARs may have preferentially promoted the growth of certain microorganisms. With increasing temperatures globally, however, further work on the impact of increased temperature on the impact of drinking water distribution system biofilm is warranted.

Another consideration is the observed temporal variability in the relative abundance of taxa in both the cast iron and cement ARs in the early stages of the study. This variability was likely influenced by both sampling frequency and the rotation through the three different ARs within each set at each subsequent collection date. However, it is important to note that even when analyzing AR-specific relative abundance over time (Supplementary Figure 4), shifting of taxa can be observed between consecutive time points. These differences in relative abundance in each AR may also suggest the presence of spatial heterogeneity of biofilm along distribution system pipe.

While DWDS pipe biofilm was only sampled at one point in time, notably, AR biofilms were different from biofilm recovered from the DWDS pipe. This may be a result of the maturity of these DWDS pipe biofilm compared to the relatively nascent annular reactors examined in the present study. In a similar laboratory-based study that examined biofilm over a 2-year period, the researchers observed a difference in reactor and DWDS biofilm (Aggarwal et al., 2018). In addition to greater biofilm age, the convergence (if ever) of the composition of the AR biofilm with the DWDS biofilm may require more realistic environmental conditions (Martiny et al., 2003; Neu et al., 2019). While best efforts were made to simulate *in situ* conditions of the DWDS, the higher temperature and low chlorine levels within the study ARs suggest that the AR biofilm may be more representative of biofilm that may be found in dead-end, stagnant zones within DWDS or in systems that have warmer water temperatures. Even though the ARs were plumbed as close as possible to the water main, similar to the island-mainland dynamics found by Ling et al. (2018), some variability may exist between the water main and the ARs as a result of neutral processes, such as migration and demographic stochasticity, or even from the impacts of biofilm on pipe between the main and the ARs.

The AR biofilm in the present study may also reflect and demonstrate the impact of modern conditions within DWDS, including the presence of pipe of various ages and material as well as modern water treatment methods. The microbial communities on the cement coupons of the ARs can provide valuable insights into the current and future impacts of replacing cast iron pipe in aging distribution systems with cement-lined pipe.

Another caveat is the reliance of this present work on sequencing data and not culture data. Fu et al. (2021) detected culturable and viable but not culturable (VBNC) waterborne pathogens in biofilm on various pipe material of a chlorinated drinking water distribution system, suggesting that pathogenic organisms in pipe biofilm may impact the safety of drinking water. Unfortunately, the present study did not investigate the presence of culturable or VBNC pathogens. Some of the organisms detected in the present study may not be viable, therefore limiting any assessment of the interactions between biofilm taxa and direct health risks of water consumption. Additionally, of note, while some opportunistic pathogens were identified in the biofilm samples, many of the identified species have previously been recovered from clinical samples in other studies but are not associated with waterborne transmission (Wilkinson et al., 1985; Best and Best, 2009; Bouam et al., 2017).

The present study focused on cast iron and cement pipe only because these materials were being used by the local utility. The results of this study allow for a comparison between biofilm on aged cast iron coupons (similar to the aging cast iron pipe in the current DWDS, especially after flushing and disinfection following main breaks), with biofilm on new, cement coupons (similar to the cement-lined replacement pipe for the DWDS). However, many DWDS globally are replacing their pipe with myriad materials, including steel, polyethylene, and PVC. Bacterial levels, diversity, and composition differ on biofilm on cast iron and PVC substrata (Norton and LeChevallier, 2000; Lin et al., 2013; Liu R. et al., 2014). Polyethylene has also been associated with biofilm development and diversity (van der Kooij et al., 2005; Yu et al., 2010). Douterelo et al. (2014) found that, while cast iron pipe sections were associated with greater biofilm development, greater diversity was recovered from polyethylene pipe sections. To better guide decisions on DWDS pipe replacement, future studies would benefit from a more comprehensive analysis of different pipe materials, such as polyethylene and PVC, under environmental conditions similar to that of a DWDS.

### Relevance and Recommendations to the Drinking Water Sector

Globally, the deterioration of highly corroded and/or aging water mains, leaks, and water main breaks have been observed in many DWDS (Bruaset and Sægrov, 2018; Folkman, 2018). Water main breaks result in the interruption of water supply, damage to surrounding property, and increased risks of pathogen intrusion (LeChevallier et al., 2003; Nygård et al., 2007). Many water mains around the world are reaching or have surpassed their life expectancy and are in great need of replacement to ensure the continuous provision of safe drinking water [American Water Works Association (AWWA), 2017; Renwick et al., 2019]. As these pipes are replaced, utilities must weigh myriad factors when choosing the pipe material for replacement pipe, including but not limited to, climate and chemical properties of the piped water and soil. Another consideration that cannot be overlooked is the impact of pipe material on chlorine levels and the survival and growth of pathogens associated with DWDS, such as *Legionella pneumophila* and *Mycobacterium avium*.

The present work indicates that cast iron pipe are associated with lower chlorine levels and greater prevalence of *Legionella* spp. In contrast, cement pipe are associated with higher chlorine levels and greater prevalence of mycobacteria, which, compared to *Legionella* spp., are known to be more difficult to control with chloramine (Taylor et al., 2000; Wang et al., 2014; Donohue et al., 2019). These results suggest that, when replacing pipe, the impact of pipe material on disinfectant residual should also be taken into consideration to curb the proliferation of *Legionella* spp. and *Mycobacterium* spp., given that some species within these genera represent opportunistic pathogens. Yet, there is no clear indication from the present study which pipe material is favorable to the wholesale prevention of opportunistic pathogen presence and instead, the results suggest that trade-offs may exist for the prevention of specific opportunistic pathogens. While the control of mycobacteria and *Legionella* spp. cannot be addressed by pipe material alone, these trade-offs may necessitate adaptive strategies tailored to each distribution system and the communities served by these systems.

Because biofilm development is inevitable in water systems, it is important to understand the drivers of microbial community composition within these water systems and the ways in which certain members of these communities may facilitate (directly or indirectly) the growth of human pathogens. For example, *L. pneumophila* can replicate through the infection of protozoan hosts that prey upon various bacteria, with a proclivity for β-proteobacteria (van der Kooij et al., 2018). This may mean that the higher prevalence of β-proteobacteria in biofilm of cast iron pipe (vs. cement pipe) may better promote the growth of *L. pneumophila*. The high β-proteobacteria prevalence on cast iron coupons in concert with the lower chlorine levels observed in the cast iron ARs of the present study may have contributed to the greater prevalence of *Legionella* spp. in the cast iron biofilm.

Furthermore, better understanding the impact of the diversity of microbial communities present on pipe of varying materials is also important to understand the prevalence of opportunistic pathogens. As previously noted, species diversity may allow biofilm within drinking water pipe to resist colonization with pathogens. Further work is necessary to determine the water quality benefits conferred by biofilm diversity and in particular, the presence of specific taxa within biofilm. However, the present results suggest that the impact of pipe material on the diversity and composition of microbial communities within biofilm should also be considered by utilities.

## Data Availability Statement

The datasets presented in this study can be found in online repositories. The names of the repository/repositories and accession number(s) can be found below: NCBI SRA BioProject, accession no: PRJNA771194.

## Author Contributions

DL and GC: writing and analysis. KK: writing. RH, SC, and DM: analysis. HM: study design, writing, and analysis. All authors contributed to the article and approved the submitted version.

## Conflict of Interest

The authors declare that the research was conducted in the absence of any commercial or financial relationships that could be construed as a potential conflict of interest.

## Publisher’s Note

All claims expressed in this article are solely those of the authors and do not necessarily represent those of their affiliated organizations, or those of the publisher, the editors and the reviewers. Any product that may be evaluated in this article, or claim that may be made by its manufacturer, is not guaranteed or endorsed by the publisher.
