# Supplementary material for: The Impact of Pipe Material on the Diversity of Microbial Communities in Drinking Water Distribution Systems
Source: Front Microbiol. 2021 Dec 21;12:779016. doi: 10.3389/fmicb.2021.779016 (PMC8724538; doi:10.3389/fmicb.2021.779016)
Supplement: Supplementary file 2 [file Data_Sheet_1.docx]

**Supplementary Material**

**
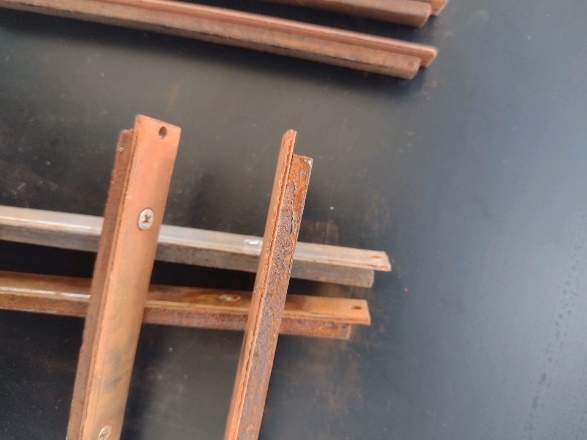

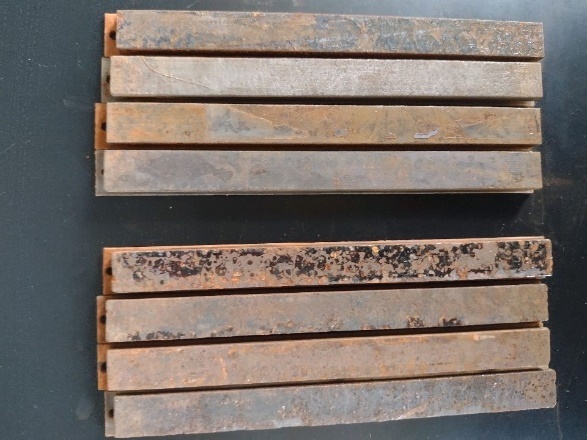
**

**Supplementary Figure S1.** Photos of the cast iron coupons used in the annular reactors.

**
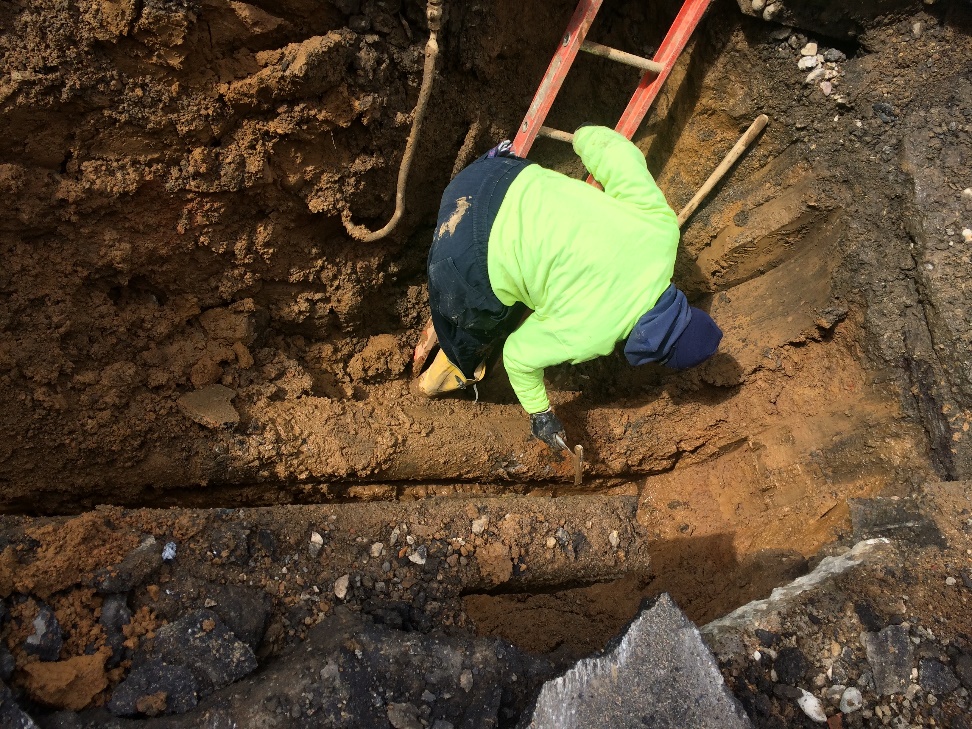

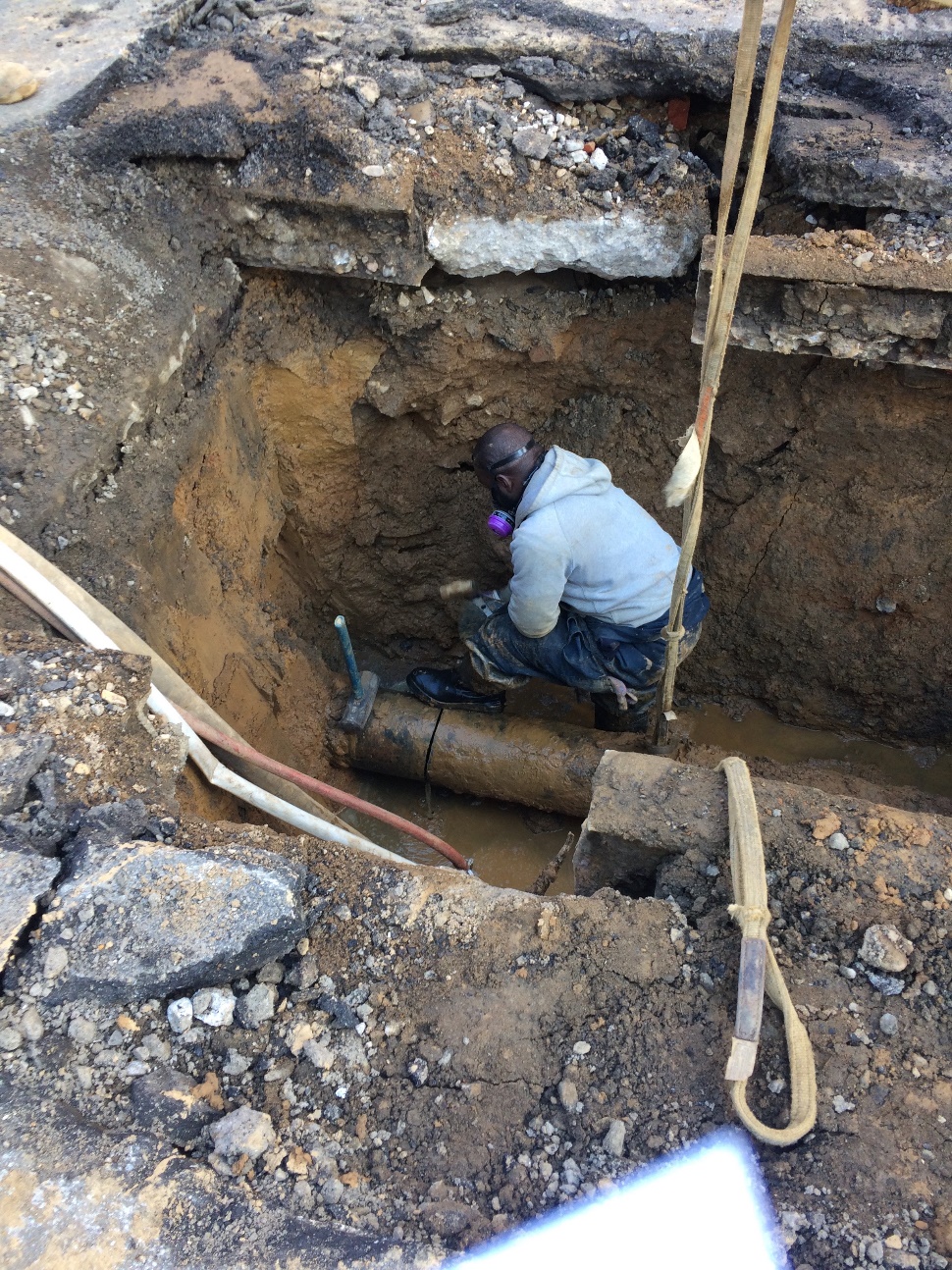
**

**Supplementary Figure S2**. Photos of the main from which distribution system (DS) pipe biofilm samples were collected during a main break on March 5, 2018.


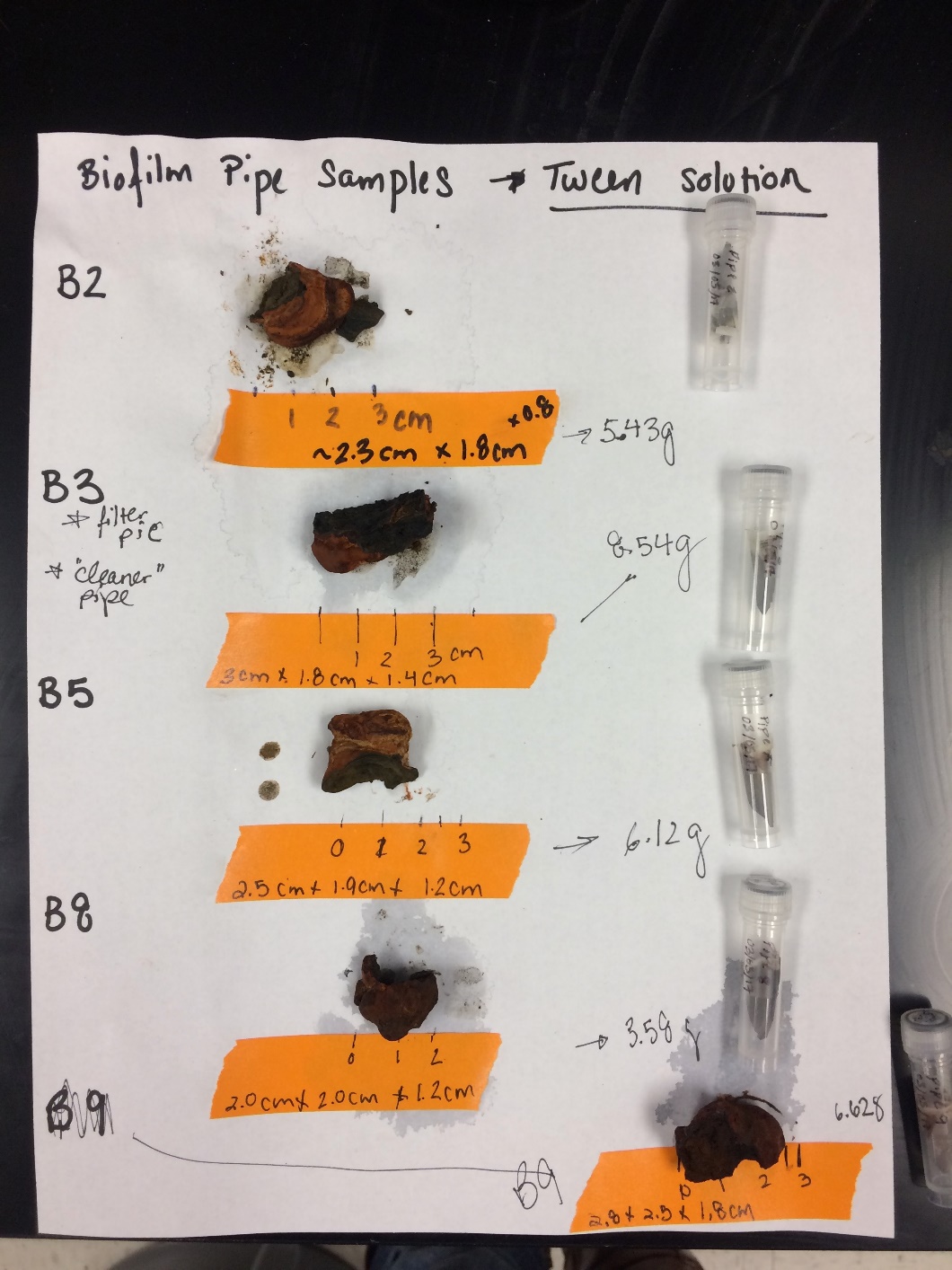


**Supplementary Figure S3.** Examples of the five samples (out of nine total) collected from the main break with the amounts (in grams) added to the 0.1% Tween-80 solution. For each sample, the dimensions (L x W x H in cm) of the sample are also displayed.

**Supplementary Text S1.** Genomic DNA was amplified using primers, 341F and 785R, targeting the V3-V4 region of the 16S rRNA gene (amplicon 460 bp).

| 341F | 5' - CCTACGGGNGGCWGCAG |
| --- | --- |
| 785R | 5' - GACTACHVGGGTATCTAATCC |

**Supplementary Text S2**. The relative abundance of *Nitrosomonas* spp. (ammonia-oxidizing bacteria) was not significantly different between cast iron and cement samples (Kruskal-Wallis χ^2^ = 3.31; p = 0.07). The relative abundance of *Nitrospira* spp. (nitrite-oxidizing bacteria) was significantly greater in cast iron samples (mean: 7.4 ± 4.5%) than in cement samples (mean: 3.0 ± 5.5%) (Kruskal-Wallis χ^2^ = 26.25; p < 0.001). Interestingly, *Nitrospiraceae* increase over time in both the cast iron and cement ARs (Figure 3) and this observation is more notable in the cement ARs. *Nitrospiraceae* may have increased in the ARs in the final months because of increased levels of chloramine in the system in the summer months (Figure 1) of the second year. It is possible that *Nitrospiraceae* was unable to robustly colonize the ARs in the early stages of biofilm development, but as the biofilm matured, *Nitrospiraceae* was able to colonize the biofilm and, with increasing temperatures and the additional chloramine administered to the drinking water distribution system, proliferate. The relative abundance of *Nitrospiraceae* in the cast iron vs. cement reactors is complicated by the cessation of cast iron AR sampling two months before the cessation of cement AR sampling; however, it is possible that the greater relative abundance in the cement ARs (as compared to the cast iron ARs) may be due to the lower microbial diversity and lower levels of competition within cement ARs.

**Supplementary Text S3**. The relative abundance of genes associated with nitrogen metabolism pathways (KEGG Orthology: ko00910) was significantly greater in cement reactors than in cast iron reactors (Kruskal-Wallis χ^2^ = 7.34; p = 0.007). The relative abundance of these genes also differed by season, with significant differences between winter and autumn, and winter and summer (Bonferroni corrected post hoc Dunn’s Test; p < 0.05). There was a significant negative association between the relative abundance of these nitrogen metabolism pathway genes and temperature (Kruskal-Wallis χ^2^ = 20.12; p = 0.01). The relative abundance of genes associated with ammonia monooxygenase (*amoA, amoB, amoC*) was significantly greater in cast iron reactors than in cement reactors (Kruskal-Wallis χ^2^ = 14.97; p < 0.001) but did not significantly differ by season (Kruskal-Wallis χ^2^ = 0.06; p = 0.996). The abundance of KEGG Orthology pathways in samples can be found in Supplementary Table S4.

Several genes associated with denitrification were identified in our samples (via analysis of MetaCyc pathways: DENITRIFICATION-PWY, PWY-7084, PWY490-3) and the relative abundance of these genes were higher in cast iron samples (mean relative abundance = 0.20 ± 0.06%) than in cement samples (mean relative abundance = 0.10 ± 0.04%) (Kruskal-Wallis χ^2^ = 44.86, p < 0.001). The relative abundance of these genes did not differ by season (Kruskal-Wallis χ^2^ = 2.19, p = 0.53). The abundance of MetaCyc pathways can be found in Supplementary Table S5.

The present study observed a significantly higher relative abundance of genes associated with pathways involving ammonia oxidation, nitrifier denitrification (ammonia to nitrite to nitric oxide, nitrous oxide, and molecular nitrogen), and nitrate reduction (associated with a reduced ferredoxin iron-sulfur cluster) in the cast iron samples. The abundance of these genes in cast iron samples may be driven by increased chloramine decay in the presence of iron (Arevalo, 2007; Westbrook and Digiano, 2009) and the subsequent prevalence of ammonia. However, when comparing all of the KEGG Orthology pathways involving nitrate metabolism, the relative abundance of all of the nitrogen metabolism-related genes combined was greater in the cement samples. This discrepancy may be due to the numerous pathways that are involved in nitrogen metabolism and thus, further work is necessary to better understand the impact of pipe material on specific functional pathways.

Analyzing the abundance of genes associated with KEGG Orthology and MetaCyc pathways is one method of elucidating the functional microbial ecology of pipe biofilm. However, it is important to note that the PICRUSt2 analyses are amplicon-based predictions of functional potential and not direct measurements of gene presence or gene expression. The growing accessibility of high-throughput sequencing—and in turn, a greater prevalence of metagenomic data on the microbiome of drinking water distribution systems—must be coupled with a better understanding of the public health and water quality implications of biofilm diversity, the relative abundance of specific taxa, and the functional composition of biofilm (as validated by downstream transcriptional analyses).

**
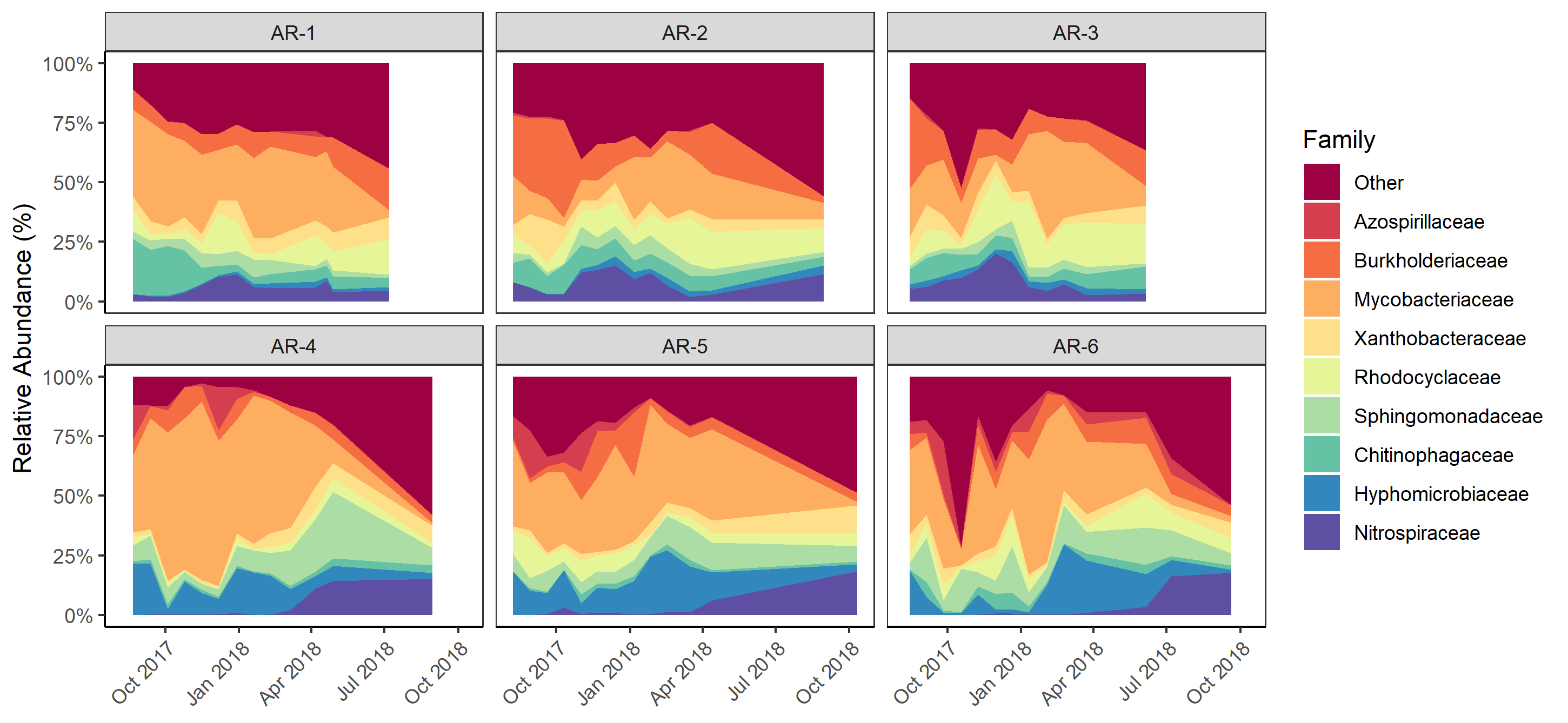
**

**Supplementary Figure S4.** Relative abundance of the most prevalent taxa in annular reactor (AR) biofilm samples over the 16-month study period. Cast iron coupons were in ARs 1-3; cement coupons were in ARs 4-6.

**
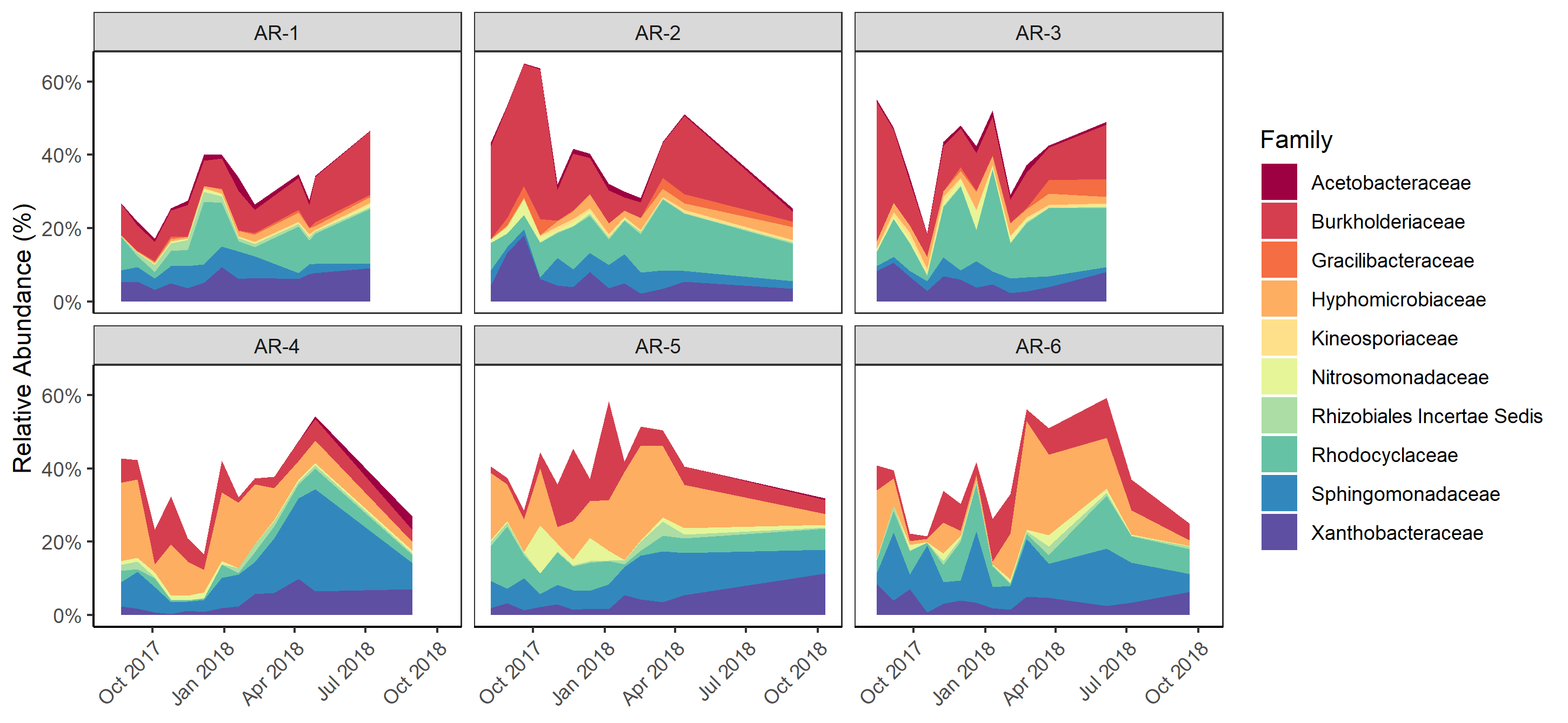
**

**Supplementary Figure S5.** Relative abundance of differentially abundant taxa in annular reactor (AR) biofilm samples over the 16-month study period as identified by Longitudinal Analysis of Composition of Microbiomes (ANCOM) analysis. Cast iron coupons were in ARs 1-3; cement coupons were in ARs 4-6.

**Supplementary Table S1**. Differences in alpha diversity and richness by pipe material using three metrics: Shannon H Index, Faith’s PD, and Observed ASVs. Kruskal-Wallis tests were used to evaluate differences by pipe material.

| Diversity/Richness metric | Cast iron  Mean (± SD) | Cement  Mean (± SD) | Kruskal-Wallis χ^2^ (p-value) |
| --- | --- | --- | --- |
| Shannon | 5.00 ± 0.41 | 4.16 ± 0.78 | 1242 (p<0.001) |
| Faith’s PD | 15.40 ± 2.88 | 13.00 ± 2.01 | 1107 (p<0.001) |
| Observed ASVs | 149.84 ± 31.35 | 114.20 ± 36.93 | 1205 (p<0.001) |

**Supplementary Table S2** – Spearman’s Rank-Order Correlation between physicochemical water quality parameters and Shannon H indices, Faith’s PD, and Observed ASVs.

| Sample Source | Parameter | Shannon | Faith’s PD | Observed ASVs |
| --- | --- | --- | --- | --- |
| Influent | Max Temp | -0.07 (0.52) | -0.47 (<0.001) | -0.37 (0.001) |
|  | Max Conductivity | -0.28 (0.01) | 0.08 (0.46) | 0.04 (0.72) |
|  | Max Chlorine | -0.25 (0.03) | -0.09 (0.42) | -0.12 (0.28) |
|  | Max Turbidity | -0.16 (0.16) | 0.19 (0.10) | 0.17 (0.15) |
|  | Mean Temp | -0.02 (0.88) | -0.50 (<0.001) | -0.40 (0.0003) |
|  | Mean Conductivity | -0.28 (0.01) | -0.01 (0.92) | -0.04 (0.76) |
|  | Mean Chlorine | 0.11 (0.33) | 0.27 (0.02) | 0.24 (0.03) |
|  | Mean Turbidity | 0.14 (0.24) | 0.35 (0.001) | 0.27 (0.02) |
| AR | Mean Temp | -0.11 (0.36) | -0.59 (<0.001) | -0.46 (<0.001) |
|  | Mean pH | -0.18 (0.12) | -0.51 (<0.001) | -0.42 (0.0002) |
|  | Mean Conductivity | -0.18 (0.12) | 0.07 (0.57) | 0.01 (0.91) |
|  | Mean Turbidity | 0.15 (0.23) | 0.02 (0.84) | 0.14 (0.24) |

**
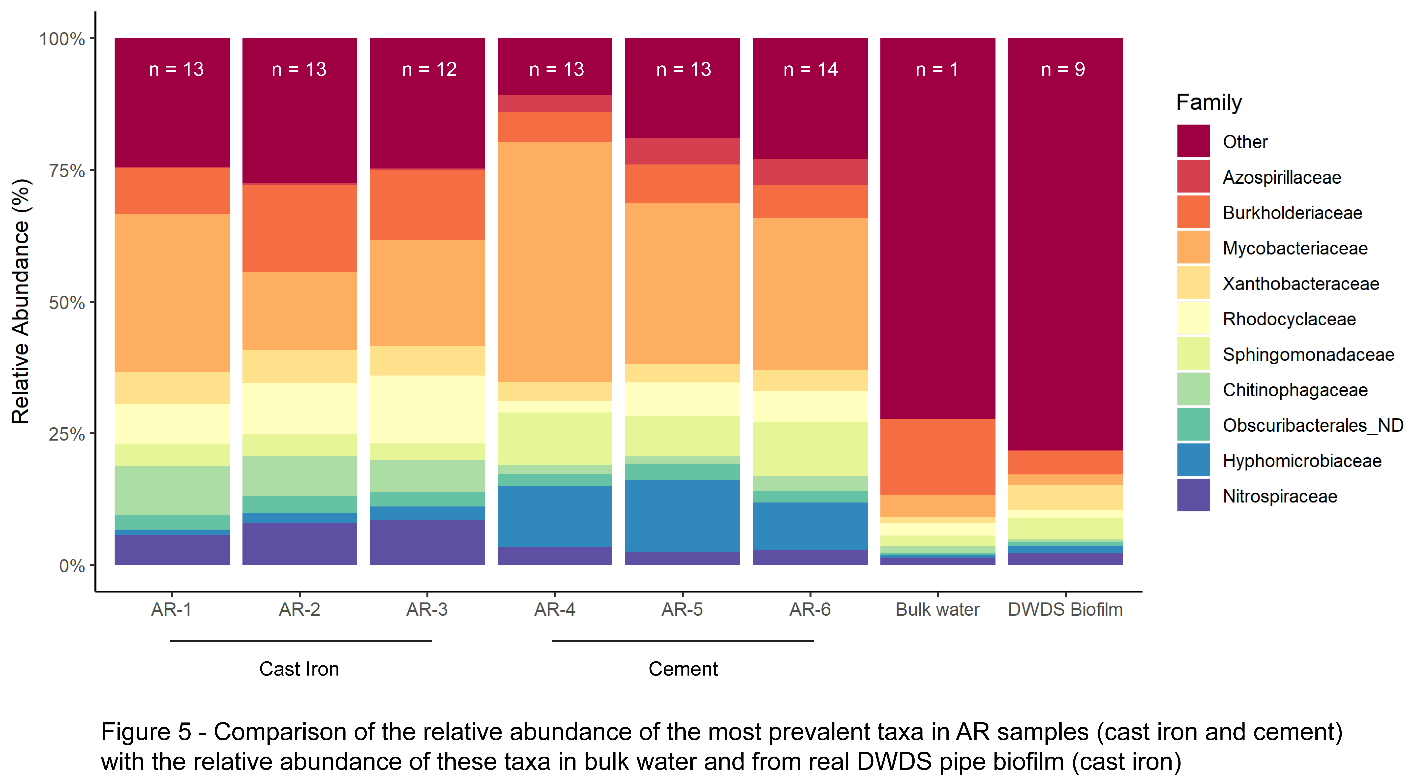
**

**Supplementary Figure S6**. Comparison of the relative abundance of the most prevalent taxa in AR samples (cast iron and cement) with the relative abundance of these taxa in bulk water and from real drinking water distribution system (DWDS) pipe biofilm (cast iron). A total of 38 cast iron coupons and 40 cement coupons were collected from ARs over the 16-month study period.

**
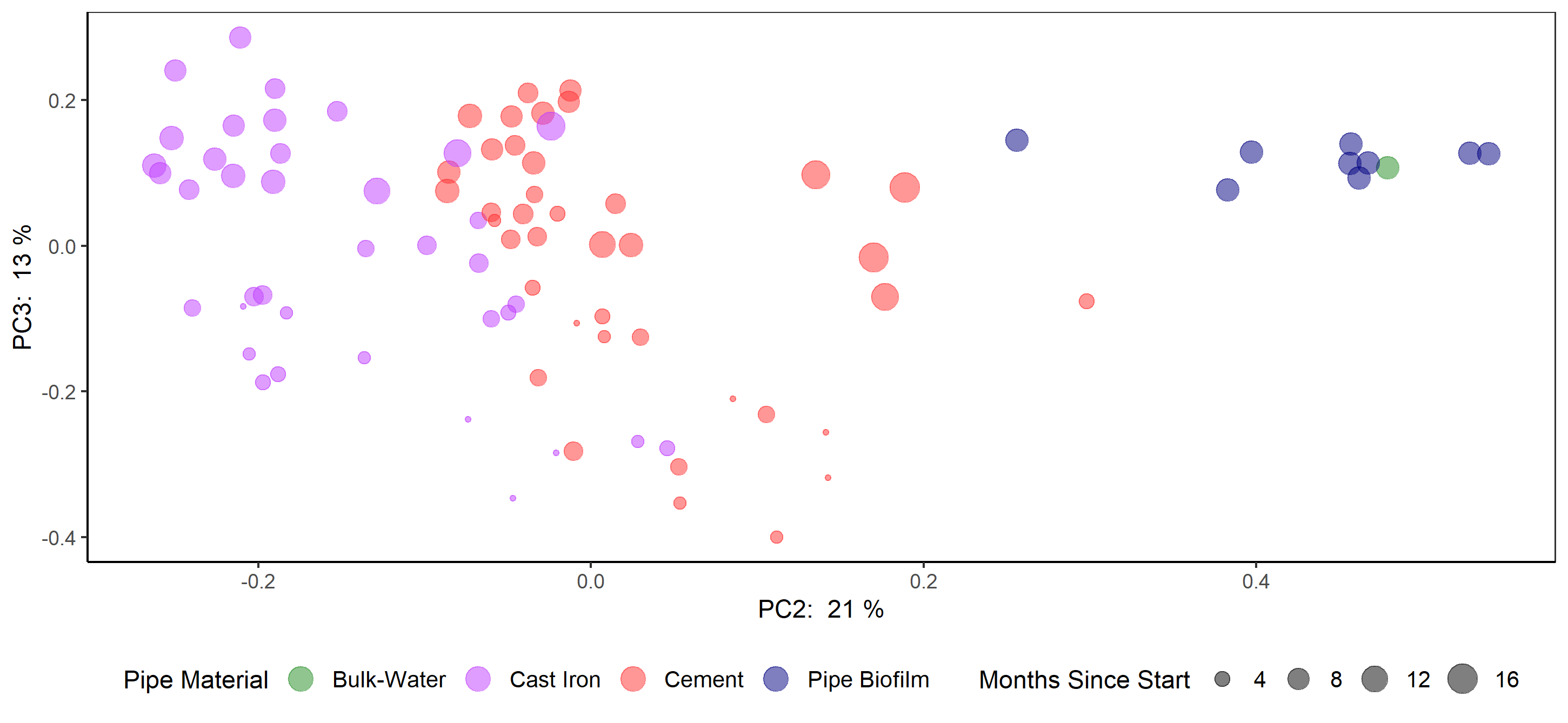
**

**Supplementary Figure S7.** Comparison of bacterial communities in annular reactors (cast iron and cement) and drinking water distribution systems (bulk water and pipe biofilm). Principal coordinate analysis (PCoA) plots of the Bray-Curtis distances according to their sample type (color) and months since start of study (size).

1. PC1 vs PC2


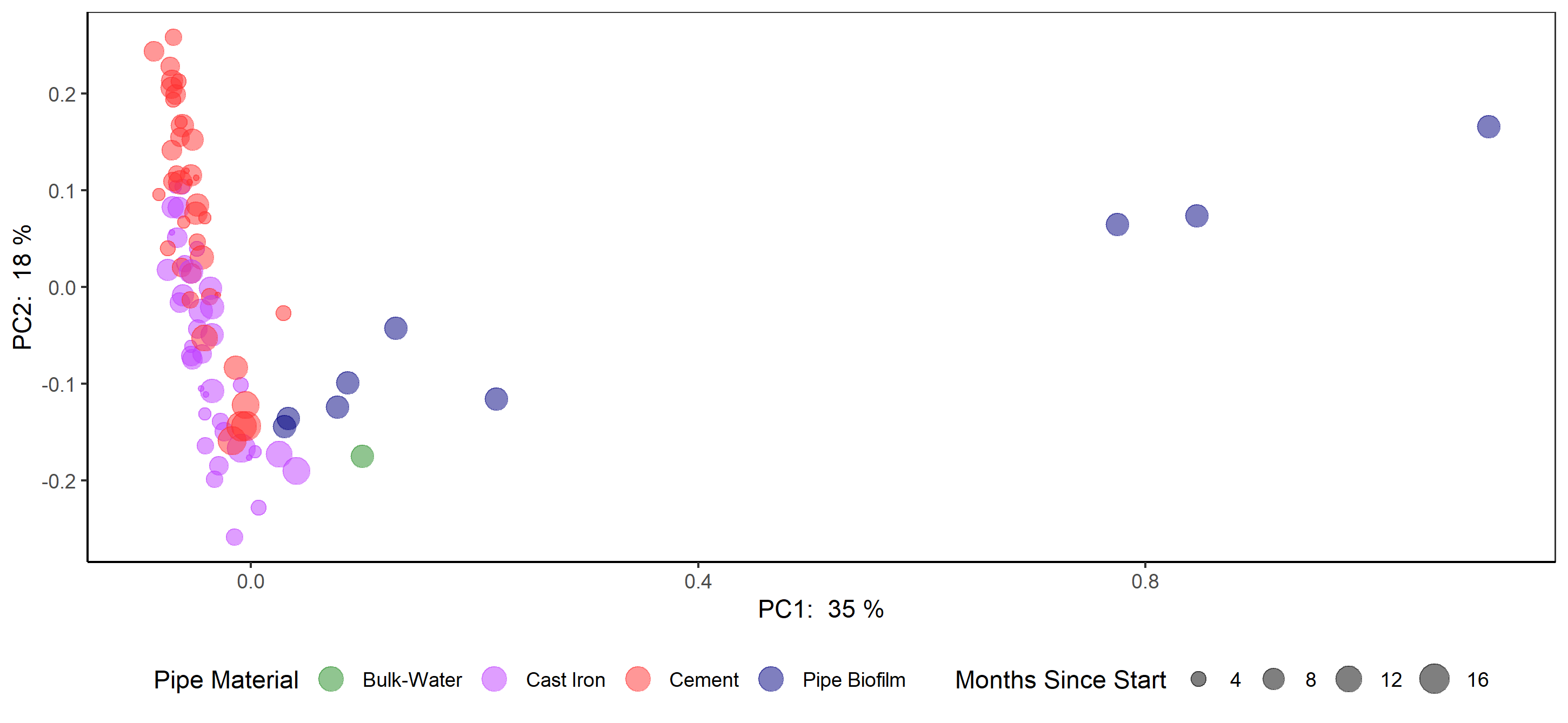


1. PC2 vs. PC3


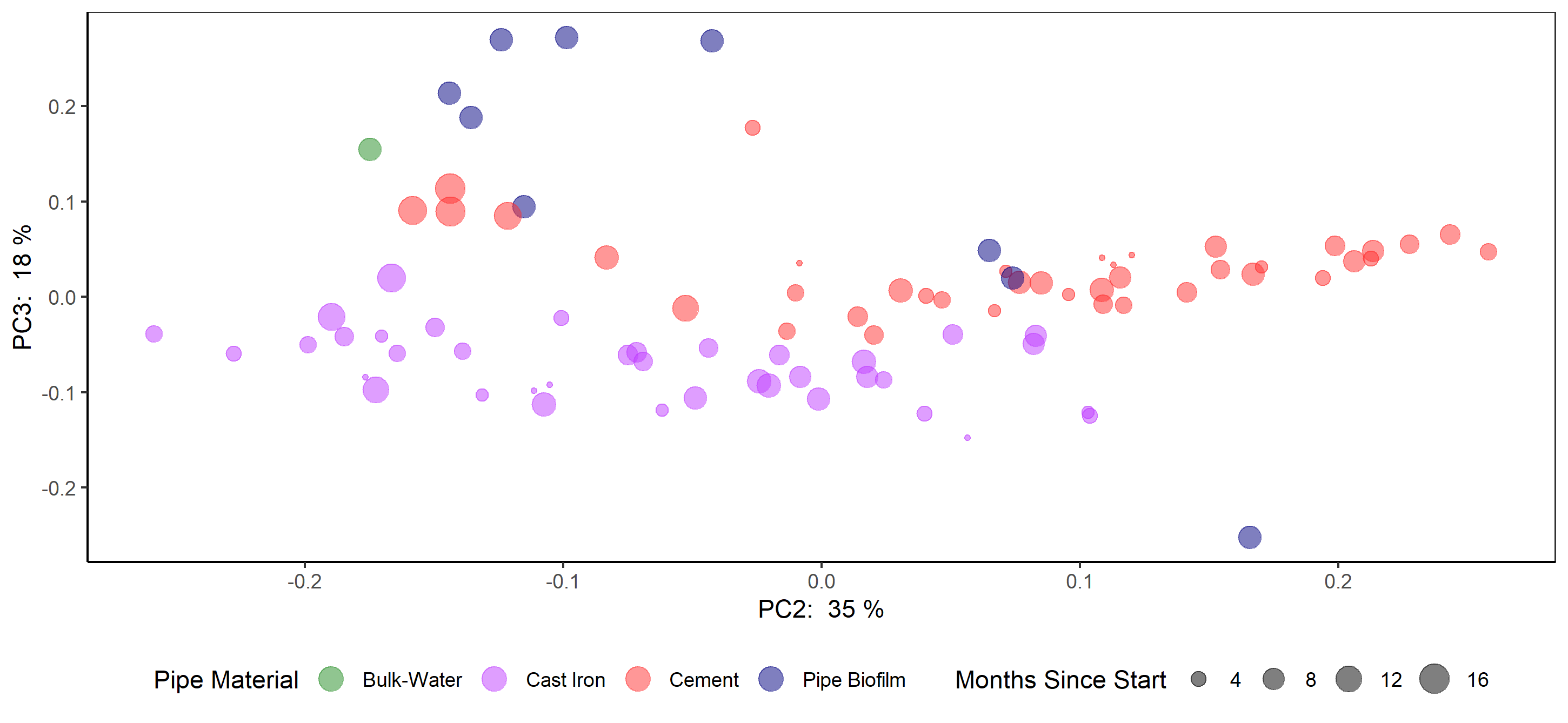


**Supplementary Figure S8.** Comparison of bacterial communities in annular reactors (cast iron and cement) and drinking water distribution systems (bulk water and pipe biofilm). Principal coordinate analysis (PCoA) plots of the weighted UniFrac distances according to their sample type (color) and months since start of study (size).

**Supplementary Table S3** – Results of PERMANOVA by a) pipe material and c) season along with the results of permutation tests of homogeneity of dispersion by b) pipe material and d) season

1. Results of PERMANOVA by pipe material

|  | Df | Sums of Squares | Mean Sqs | F Model | R2 | Pr(>F) |
| --- | --- | --- | --- | --- | --- | --- |
| Season | 1 | 4.53 | 4.53 | 24.20 | 0.24 | 0.001 |
| Residuals | 76 | 14.24 | 0.19 |  | 0.76 |  |
| Total | 77 | 18.77 |  |  | 1.00 |  |

1. Results of permutation test of homogeneity of dispersion by pipe material

|  | Df | Sum Sq | Mean Sq | F | N. Perm | Pr (>F) |
| --- | --- | --- | --- | --- | --- | --- |
| Groups | 1 | 0.01 | 0.01 | 0.91 | 999 | 0.34 |
| Residuals | 76 | 0.88 | 0.01 |  |  |  |

1. Results of PERMANOVA by season

|  | Df | Sums of Squares | Mean Sqs | F Model | R2 | Pr(>F) |
| --- | --- | --- | --- | --- | --- | --- |
| Season | 3 | 1.89 | 0.63 | 2.76 | 0.10 | 0.001 |
| Residuals | 74 | 16.88 | 0.23 |  | 0.90 |  |
| Total | 77 | 18.77 |  |  | 1.00 |  |

1. Results of permutation test of homogeneity of dispersion by season

|  | Df | Sum Sq | Mean Sq | F | N. Perm | Pr (>F) |
| --- | --- | --- | --- | --- | --- | --- |
| Groups | 3 | 0.18 | 0.06 | 9.92 | 999 | 0.001 |
| Residuals | 74 | 0.46 | 0.01 |  |  |  |

**Supplementary Table S4** – Relative abundance of KEGG Orthology Pathways in annular reactor (AR), distribution system pipe biofilm (DS_Pipe), and distribution system bulk water samples (DS_BW). AR 1-3 had cast iron coupons while AR 4-6 had cement coupons. Sample names include the date of sample collection in the following format: Month-Day-Year.

(See: KO-Abundance.csv)

**Supplementary Table S5** – Relative abundance of MetaCyc Pathways in annular reactor, distribution system pipe biofilm, and distribution system bulk water samples. ARs 1-3 had cast iron coupons while ARs 4-6 had cement coupons. Sample names include the date of sample collection in the following format: Month-Day-Year.

(See: MC-Abundance.csv)
